# Coral endosymbionts (Symbiodiniaceae) emit species-specific volatilomes that shift when exposed to thermal stress

**DOI:** 10.1038/s41598-019-53552-0

**Published:** 2019-11-22

**Authors:** Caitlin A. Lawson, Malcolm Possell, Justin R. Seymour, Jean-Baptiste Raina, David J. Suggett

**Affiliations:** 1 Climate Change Cluster (C3), University of Technology Sydney, Sydney, Australia; 2 School of Life and Environmental Sciences, University of Sydney, Sydney, Australia

**Keywords:** Biogeochemistry, Biochemistry, Ocean sciences, Marine chemistry, Analytical biochemistry, Mass spectrometry

## Abstract

Biogenic volatile organic compounds (BVOCs) influence organism fitness by promoting stress resistance and regulating trophic interactions. Studies examining BVOC emissions have predominantly focussed on terrestrial ecosystems and atmospheric chemistry – surprisingly, highly productive marine ecosystems remain largely overlooked. Here we examined the volatilome (total BVOCs) of the microalgal endosymbionts of reef invertebrates, Symbiodiniaceae. We used GC-MS to characterise five species (*Symbiodinium linucheae*, *Breviolum psygmophilum*, *Durusdinium trenchii*, *Effrenium voratum*, *Fugacium kawagutii*) under steady-state growth. A diverse range of 32 BVOCs were detected (from 12 in *D*. *trenchii* to 27 in *S*. *linucheae*) with halogenated hydrocarbons, alkanes and esters the most common chemical functional groups. A thermal stress experiment on thermally-sensitive *Cladocopium goreaui* and thermally-tolerant *D*. *trenchii* significantly affected the volatilomes of both species. More BVOCs were detected in *D*. *trenchii* following thermal stress (32 °C), while fewer BVOCs were recorded in stressed *C*. *goreaui*. The onset of stress caused dramatic increases of dimethyl-disulfide (98.52%) in *C*. *goreaui* and nonanoic acid (99.85%) in *D*. *trenchii*. This first volatilome analysis of Symbiodiniaceae reveals that both species-specificity and environmental factors govern the composition of BVOC emissions among the Symbiodiniaceae, which potentially have, as yet unexplored, physiological and ecological importance in shaping coral reef community functioning.

## Introduction

Photosynthetic organisms, ranging from complex vascular plants to single celled microalgae, are major producers of biogenic volatile organic compounds (BVOCs), which can represent up to 10% of fixed carbon and play numerous physiological and ecological roles^1,2^. BVOCs are exceptionally diverse and highly reactive, with chemical lifetimes ranging from minutes (e.g. β-caryophyllene) to months (e.g. acetone)^3^, and can be synthesised as by-products of metabolic pathways^4^ or be produced to maintain metabolic homeostasis^3^. The roles played by these compounds are multifaceted, from protection against abiotic stress^5,6^ and pathogens^7,8^, to chemical signalling^9–12^.

Terrestrial tropical ecosystems are well recognised “hotspots” of global BVOC emissions^13^, but growing evidence also highlights the role of tropical marine ecosystems, such as coral reefs^14,15^ as major sources of BVOC emissions. Reef-building corals emit the highest recorded concentrations of the sulphur gas dimethyl sulfide (DMS; up to 18.7 µM reported in coral mucus)^16,17^, a compound involved in climate regulation^18^, coral stress response^5,19^ and functioning as an infochemical^20,21^. Furthermore, the presence of acetone and dichloromethane was recently identified in reef seawater samples, indicating BVOCs produced in coral reefs are likely to be a complex mixture^22^. In addition, cultures of the dinoflagellate endosymbionts of reef-building corals (Family: Symbiodiniaceae) can produce DMS^19,23,24^ and isoprene^25^. However, these observations are derived from targeted quantifications of specific BVOCs and likely represent only a small fraction of the compounds that Symbiodiniaceae can produce.

Emission of BVOCs from corals are thought to largely originate from Symbiodiniaceae due to the large quantities of carbon and metabolites they translocate^26,27^. Metabolic coupling between coral host and algal endosymbionts is critical for viable coral reef functioning – whereby the Symbiodiniaceae drive coral productivity, but can also govern susceptibility of their host to stressors by controlling the exchange of nutrients^28^. Recent studies have moved towards identifying the functional diversity amongst the Symbiodiniaceae to determine key traits of resistance to stress^29,30^, which is critical to understand their susceptibility to rising seawater temperatures. Whilst different metabolic traits and hence diagnostic metabolites appear central in governing this functional diversity^31^, the role of BVOCs remains largely unexplored.

The assortment of BVOCs produced by an organism has recently been termed the ‘volatilome’^32,33^. Assessments of volatilomes, or volatilomics, is widely used in medical research, where the approach has been used to diagnose patient health via bio-markers in exhaled breath^34^. More recently, volatilomics has also been used to quickly distinguish plant strains and to successfully monitor long-term plant health^35^, with this approach showing promise for the development of new non-invasive monitoring tools of taxonomic and functional diversity in aquatic ecosystems^33^. Indeed, volatilomics may be particularly useful in connecting ecological processes and biogeochemical cycles in the ocean, since emissions of BVOCs from photosynthetic organisms can have a strong influence on atmospheric chemistry by increasing local cloud albedo or the residence time of greenhouse gases^36,37^. Within this context, characterising the volatilome of highly productive, habitat forming marine organisms should enable more accurate modelling of the impact of tropical BVOCs on climate regulation^2,38^.

Here we provide the first characterisation of the volatilome of different Symbiodiniaceae species (spanning five genera) to determine whether and how volatile metabolite signatures are conserved across divergent taxa, and to identify volatiles that may be involved in previously overlooked physiological processes, ecosystem interactions and atmospheric cycling. In addition to this screening across-strains, we also examined the volatilomes of two Symbiodiniaceae strains with different thermal tolerance thresholds under conditions of heat stress, to investigate the extent of thermally-induced changes in the volatilome and identify BVOCs potentially involved in Symbiodiniaceae thermal tolerance.

## Results

### Initial screening experiment

**Figure 1:**
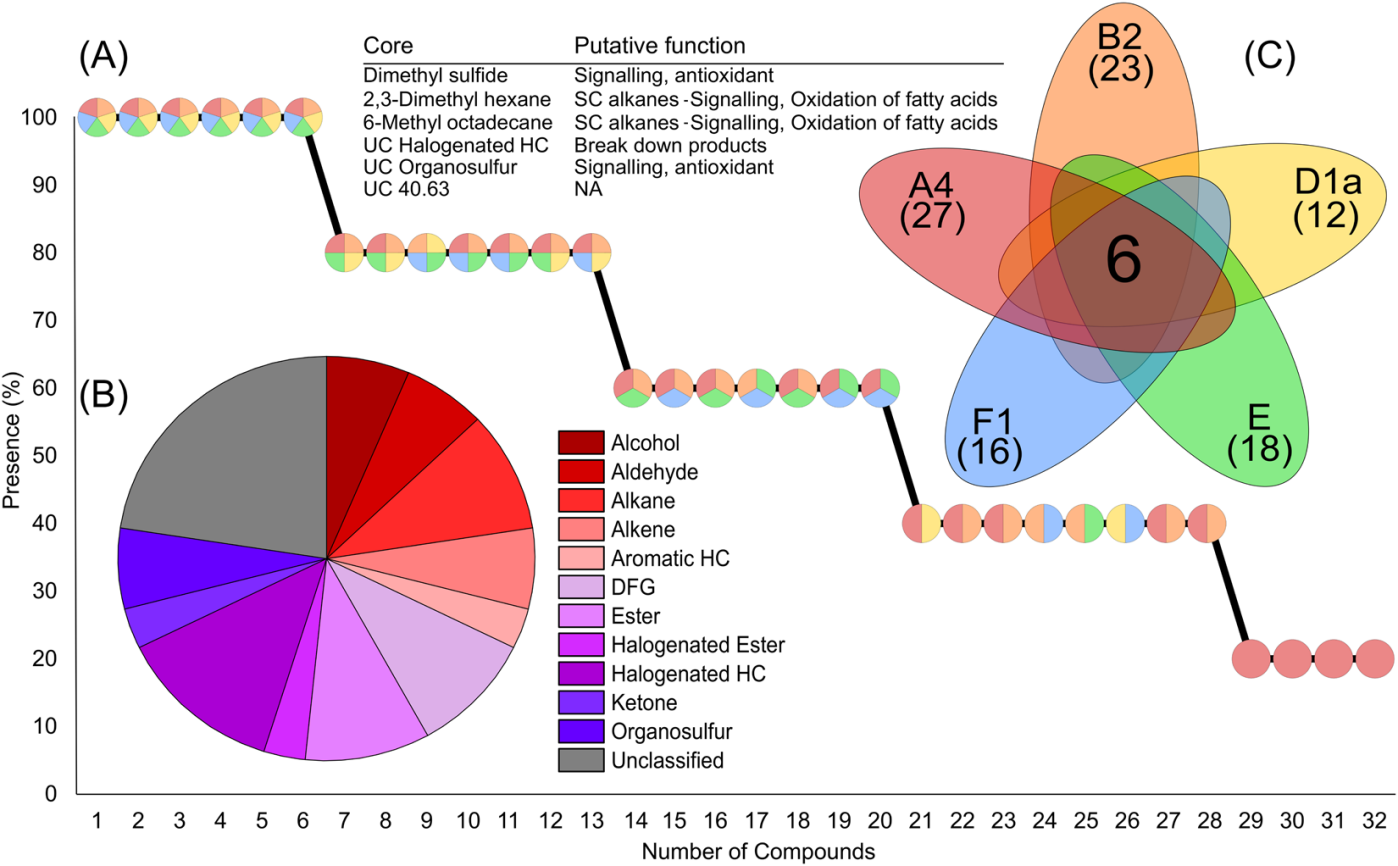
(**A**) Number of compounds detected across all Symbiodiniaceae isolates (present in at least 2 out of the 3 biological replicates), each pie-chart represents a compound and the colours denote which isolates the compound is present in. (**B**) The proportion of chemical functional groups across the 32 detected compounds from five Symbiodiniaceae species. (**C**) Venn style diagram^85^ showing the six core volatiles and their putative functions, as well as the number of compounds detected in each species (*Symbiodinium linucheae* (A4, red), *Breviolum psygmophilum* (B2, orange), *Durusdinium trenchii* (D1a, yellow), *Effrenium voratum* (E, green) and *Fugacium kawagutii* (F1, blue)). HC: hydrocarbon, DFG: diverse functional groups, SC: short chain, UC: unclassified (the number following UC indicates the retention time of the compound if the chemical functional group could not be determined).

A total of 32 BVOCs were detected across *Symbiodinium linucheae*, *Breviolum psygmophilum*, *Durusdinium trenchii*, *Effrenium voratum* and *Fugacium kawagutii*, following blank subtraction and quality control (Table S1). Of these, 50% were successfully identified and a further 28% could be assigned to functional chemical groups. Six compounds were present in at least two out of three replicates in all species tested and defined as core components of the Symbiodiniaceae volatilome (Fig. 1A). These included DMS, 2,3-dimethyl hexane, 6-methyl octadecane and three compounds that could not be fully identified (including a halogenated hydrocarbon, an organosulfur compound and an unclassified compound (UC; UC40.63) (Fig. 1A). Excluding unclassified compounds (22.6%), halogenated hydrocarbon compounds constituted the largest functional group (12.9%), followed by alkanes and esters (both 9.7%) (Fig. 1B).

**Figure 2:**
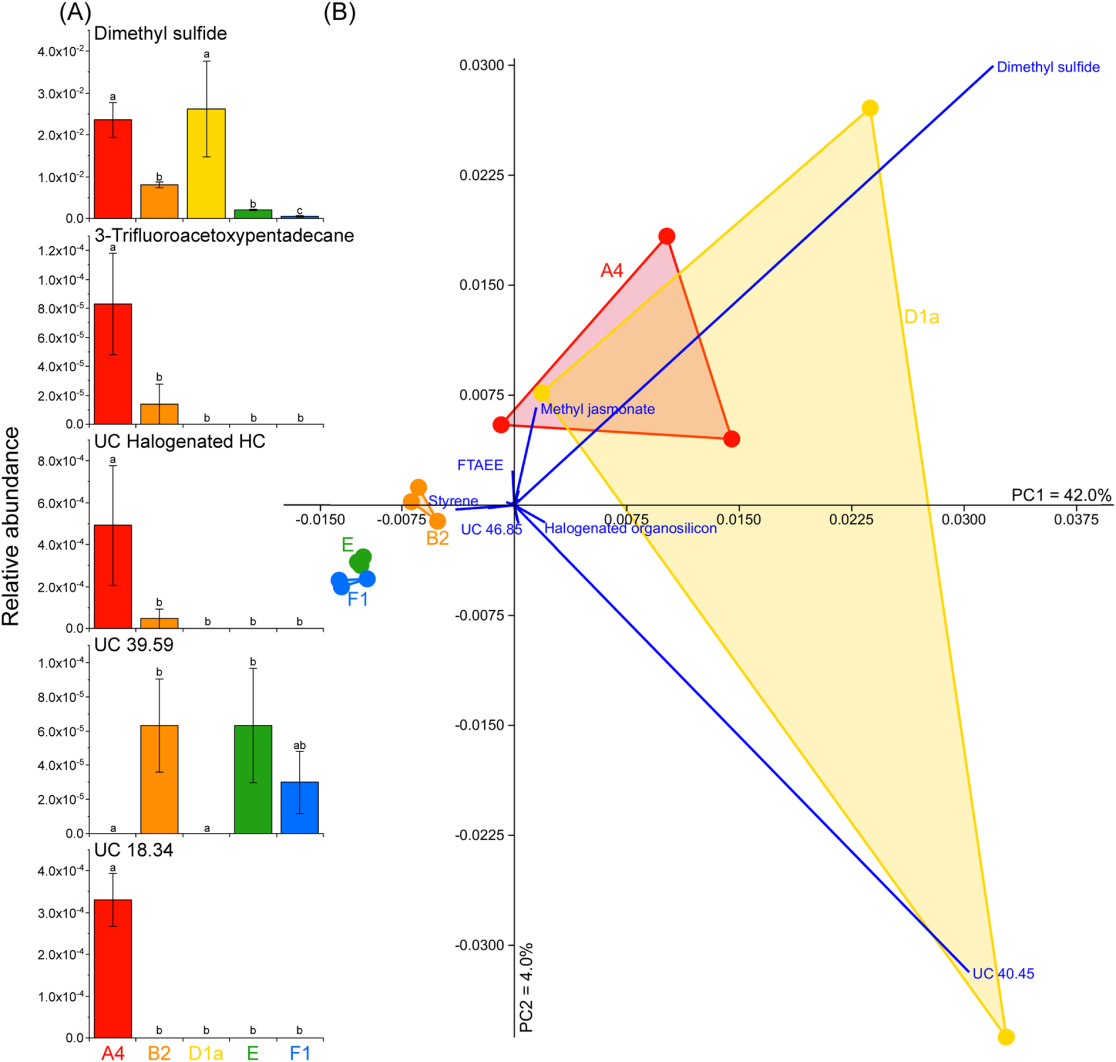
(**A**) Normalised relative abundance (mean ± standard error) for the five compounds that differed significantly between species (ANOVA, P < 0.01, Metaboanalyst 4.0^84^), significant differences are annotated on the plots. (**B**) Principal Component Analysis (PCA) of the volatilomes of *Symbiodinium linucheae* (A4, red), *Breviolum psygmophilum* (B2, orange), *Durusdinium trenchii* (D1a, yellow), *Effrenium voratum* (E, green) and *Fugacium kawagutii* (F1, blue). Bootstrap N = 1000. HC = Hydrocarbon, UC = Unclassified (the number following UC indicates the retention time of the compound if the chemical functional group could not be determined), FTAEE = 4-fluoro-3-trifluoromethylbenzoic acid eicosyl ester.

Four BVOCs were unique to *S*. *linucheae*, including 3-trifluoroacetoxypentadecane, squalene, an unknown halogenated hydrocarbon and a compound that could not be identified to functional chemical group (UC18.34). None of the volatilomes associated with any of the other Symbiodiniaceae strains comprised any unique BVOCs. The most BVOCs were detected in *S*. *linucheae* (27), followed by *B*. *psygmophilum* (23), *E*. *voratum* (18), *F*. *kawagutii* (16) and *D*. *trenchii* (12) (Fig. 1C). The abundance of five compounds differed significantly between species (one-way ANOVA; P < 0.01) – these included DMS, 3-trifluoroacetoxypentadecane, UC18.34, UC39.59 and an unclassified halogenated hydrocarbon (Fig. 2A). Principal component analysis (PCA) revealed tight groupings of *B*. *psygmophilum*, *E*. *voratum* and *F*. *kawagutii* volatilomes, while the *S*. *linucheae* and *D*. *trenchii* volatilomes overlapped, potentially due to higher variation between replicates (Fig. 2B). The variance in the principal component analysis was strongly influenced by DMS (loading score on PC1 axis = 0.72), UC40.45 (loading score on PC1 = 0.68), methyl jasmonate (loading score on PC1 = 0.03, loading score on PC2 = 0.15) and styrene (loading score on PC1 = −0.09) (Fig. 2B).

### Thermal stress experiment

**Figure 3:**
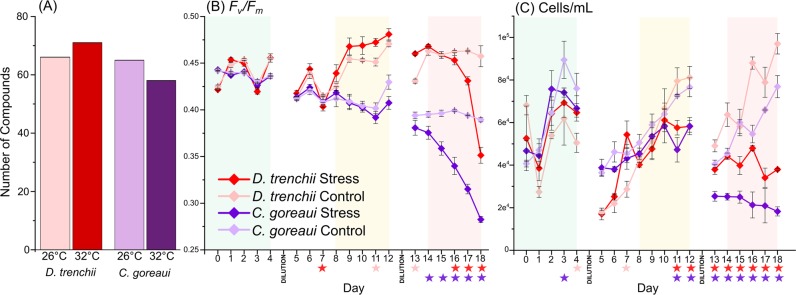
Total number of compounds (present in at least two out of three replicates) detected in *Durusdinium trenchii* and *Cladocopium goreaui* control and stress treatments (**A**). Photosynthetic health *(F*_*v*_*/F*_*m*_*)* (**B**) and cell density (cell/mL) (**C**) of *D*. *trenchii* and *C*. *goreaui* throughout the thermal stress experiment. Significant differences (P < 0.05; Kruskal Wallis; IBM SPSS version 25) between treatments are indicated with a star, the star colour indicates the significantly decreased treatment. Shading on panels (**B**) and (**C**) indicates the time period the treatment cultures were held at 26 °C (green), 30 °C (yellow) and 32 °C (red), controls were held at 26 °C throughout, see Fig. S1 for full temperature profile. All error bars are standard error.

**Figure 4:**
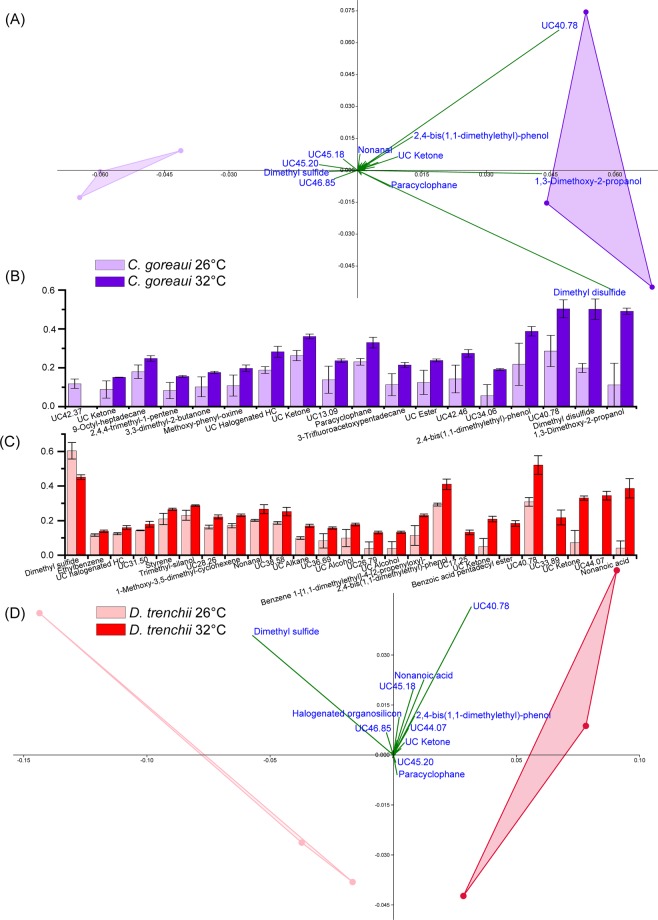
Principal Component Analysis (PCA) of *Cladocopium goreaui* (**A**) (component 1 = 62.1% and component 2 = 29.6% of variance) and *Durusdinium trenchii* (**D**) (component 1 = 74.5% and component 2 = 17.7% of variance) during the thermal stress assay experiment (PAST; Bootstrap N = 1000). Fourth root normalised abundance of compounds that varied significantly between control (26 °C) and treatment (32 °C) (P < 0.05; Kruskal Wallis; IBM SPSS version 25) are shown for *C*. *goreaui* (**B**) and *D*. *trenchii* (**C**). All error bars are standard error. HC = Hydrocarbon, UC = Unclassified (the number following UC indicates the retention time of the compound if the functional group could not be determined).

Both the heat-tolerant *D*. *trenchii* and heat-sensitive *C*. *goreaui* species exhibited a decrease in photochemical efficiency (*F*_*v*_/*F*_*m*_, dimensionless) and cell density during the thermal stress experiment, but the more thermally sensitive strain *C*. *goreaui* exhibited significant (Kruskal-Wallis, P < 0.05) declines in *F*_*v*_/*F*_*m*_ two days earlier than *D*. *trenchii* (Fig. 3B,C). A total of 72 BVOCs were detected in this stress experiment after quality control, following thermal stress there was a significant shift in volatilome composition. The relative abundance of 18 compounds differed significantly between treatment and control in *C. goreaui* (P < 0.05; see Table S2 for full list of significant differences). This shift was also reflected in a decrease in the total number of compounds detected during stress (65 to 58; present in at least two out of three replicates; Fig. 3A). Conversely, more compounds were detected in *D*. *trenchii* during stress (66 to 71; Fig. 3A) and 25 BVOCs differed significantly (P < 0.05; Table S2) between treatments. Only four compounds (2,4-bis(1,1-dimethylethyl)-phenol, two UC ketones and UC40.78) that differed significantly between treatments were shared by both species during stress (Fig. 4B,C). DMS was recorded in lower amounts under thermal stress in *D*. *trenchii* and UC42.37 became undetectable in *C*. *goreaui* during thermal stress (Fig. 4B,C). All other significantly different compounds were measured in higher quantities during thermal stress (Fig. 4B,C). Of the compounds that differed significantly between control and heat stress treatment in *C*. *goreaui*, 1,3-dimethoxy-2-propanol and dimethyl disulfide (DMDS) showed the largest increase (94.94% and 98.52%, respectively) under stress (Fig. 4B). In *D*. *trenchii*, nonanoic acid and UC44.07 exhibited the largest increases (99.85% and 100%, respectively) in the heat stress treatment (Fig. 4C). Five compounds were only detected under thermal stress conditions: benzoic acid pentadecyl ester, UC44.07, UC33.89 and UC17.25 (Fig. 4C). PCA visualised the strong differentiation between the control and thermal stress volatilomes of *C*. *goreaui* (Fig. 4A), the principal components in this PCA were influenced by DMDS (PC1 loading = 0.66), UC40.78 (PC1 loading = 0.52) and 1,3-dimethoxy-2-propanol (PC1 loading = 0.48), which were all detected in higher levels during thermal stress (Fig. 4A). PCA also revealed clear distinction between control and stress treatments in *D*. *trenchii*, whereby the principal components in the *D*. *trenchii* PCA (Fig. 4D) were largely influenced by UC40.78 (PC1 loading = 0.46), nonanoic acid (PC1 loading = 0.18) and a stronger negative forcing from DMS (PC1 loading = −0.84).

## Discussion

The multifaceted biological and ecological functions of BVOCs can influence ecosystem resilience^2^, and therefore understanding the roles of these compounds in shaping ‘healthy’ functioning of threatened ecosystems such as coral reefs is particularly important^33^. To contribute to an enhanced understanding of coral reef volatilomics, we performed the first characterisation of the volatilome of the coral endosymbionts Symbiodiniaceae, which revealed a substantial diversity of BVOC production. Our study screened species spanning a wide range of Symbiodiniaceae genera^39^, demonstrating that key species produce far more BVOCs than the iconic dimethyl sulfide. This diverse pool of volatiles included halogenated hydrocarbons, alkanes and esters among the most commonly detected compounds.

By screening five different species (spanning five genera), *S*. *linuchae*, *B*. *psygmophilum*, *D*. *trenchii*, *E*. *voratum* and *F*. *kawagutii*, we detected 32 different BVOCs. This diversity of BVOCs is consistent with a recent screening of three species of marine macroalgae (*Ulva prolifera*, *Ulva linza* and *Monostroma nitidum*), which identified 41 volatile compounds^40^. Similarly to our study, alkanes, alkenes, ketones, aldehydes, sulphur compounds, alcohols and esters were detected in the macroalgae studied^40^. However, unlike macroalgae, Symbiodiniaceae also produced halogenated hydrocarbons, and only benzaldehyde (known to be involved in signalling amongst insects^41^) was identified in both Symbiodiniaceae and macroalgae.

Of the 32 volatile compounds detected in this study, six were present in all five Symbiodiniaceae species and were therefore defined as core volatiles. Recognising the ubiquitous nature of certain volatiles is a first step in understanding the potential ecological relevance of compounds produced by this important family of microalgae. We defined DMS, 2,3-dimethyl hexane, 6-methyl octadecane, an UC halogenated hydrocarbon, UC organosulfur and UC40.63 as core volatiles. Two of these compounds, 2,3-dimethyl hexane and 6-methyl octadecane, are both short chain alkanes, which may have originated from the oxidation of fatty acids. The inclusion of DMS in the core set of volatiles produced by all Symbiodiniaceae genera examined here adds weight to the importance and ubiquitous nature of this compound in these organisms. However, it is notable that DMS relative abundance varied substantially between Symbiodiniaceae species, with significantly more DMS detected in *S*. *linucheae* and *D*. *trenchii* than all other species, while significantly less DMS was detected in *F*. *kawagutii* compared to all other species. Both *S*. *linucheae* and *D*. *trenchii* are known to be more heat resistant species^29,30,42^, potentially suggesting that the high amounts of DMS detected under steady state conditions positively influence their resilience, allowing these species to have a larger existing pool of antioxidants^19,43^.

In addition to DMS, differences between Symbiodiniaceae volatilomes were largely driven by the relative abundance of UC40.45, methyl jasmonate, styrene and 4-fluoro-3-trifluoromethylbenzoic acid eicosyl ester. Methyl jasmonate is ubiquitous in higher plants and is an important signalling molecule that regulates plant development and also plays a role in defence against biotic (e.g. herbivory) and abiotic (e.g. heat) stresses^44^. Methyl jasmonate was detected in all replicates of *S*. *linucheae*, and given previous observations in higher plants, this BVOC might be involved in the thermal resistance of this Symbiodiniaceae species. The function of the other two molecules, styrene and 4-fluoro-3-trifluoromethylbenzoic acid eicosyl ester, remain uncertain but styrene has previously been reported in higher plants^45^.

This study identified multiple halogenated compounds however, only three were fully classified (1,2-dichloropropane, 3-trifluoroacetoxypentadecane & 4-fluoro-3-trifluoromethylbenzoic acid eicosyl ester). Halogenated compounds are of particular importance as they can degrade atmospheric ozone^46^, they are known to be produced naturally^47^ and have previously been reported from marine microalgae^48^. For example, three tropical microalgae (*Amphora* sp., *Synechococcus* sp. & *Parachlorella* sp.) can produce a range of iodinated and brominated compounds (methyl iodide, bromoform, dibromomethane, dibromochloromethane, and chloroform), with production shown to be species-specific and growth-phase dependent^48^, highlighting the importance of the physiological state of the cell for volatile emissions. Other examinations of a suite of common phytoplankton (*Calcidiscus leptoporus*, *Emiliania huxleyi*, *Phaeodactylum tricornutum*, *Chaetoceros neogracilis* and *Dunaliella tertiolecta*) demonstrated that all tested species emitted chloromethane, bromoform, bromomethane, chlorobenzene and dichlorobenzene^49^. Our understanding of the function of halogenated compounds remains in its infancy, with current hypotheses suggesting the tight coupling of their production with oxidative processes (potentially formed as side products during the breakdown of reactive oxygen species)^50,51^. Halogenated compounds are also thought to sometimes function as ‘infochemicals’, with studies on macroalgae demonstrating that bromoform supports the alga’s defence by functioning as an antimicrobial^52–54^. We observed that two halogenated compounds, including 3-trifluoroacetoxypentadecane and another unclassified halogenated BVOC, differed significantly between Symbiodiniaceae species and notably seem to co-occur (Correlation: 0.73, P Value = 0.002, Pearson R, Metaboanalyst4.0^55^), with significantly higher levels of both compounds present in *S*. *linucheae* compared to all other species tested. 3-trifluoroacetoxypentadecane has previously been shown to have antimicrobial properties^56^, however, far more work is needed to accurately define the function of these halogenated compounds in Symbiodiniaceae.

This initial screening of a range of Symbiodiniaceae species has demonstrated the diversity and species-specific nature of the volatilome. Despite this diversity, a consistent core emerged, suggesting that some compounds have a conserved role across species. While appreciating these potential roles is important, most natural systems rarely remain in steady-state conditions for prolonged amounts of time. Currently, marine systems are experiencing an increase in the frequency and severity of harmful thermal stress events. How corals respond to heat-wave induced bleaching and mortality is often influenced by the thermal tolerance of their endosymbiotic algae (Symbiodiniaceae)^28^. A number of physiological traits appear to differentiate stress tolerant-versus-susceptible species (or genetic variants) of Symbiodiniaceae, including maintaining integrity of photosynthetic constituents^30,57^ and upregulation of pathways to ‘detoxify’ organelles^58^. However, the effect of thermal stress on the Symbiodiniaceae volatilome had not been explored until now.

With the onset of thermal stress, a larger number of BVOCs were detected in the thermally tolerant *D*. *trenchii*, while the thermally sensitive *C*. *goreaui* produced fewer compounds relative to control conditions. The ability to synthesise specific compounds under stress could be involved in the thermal tolerance of *D*. *trenchii*, as ‘*de novo’* BVOC synthesis has been previously observed in higher plants during abiotic stresses^4,6,59^. During thermal stress, concomitantly with a decrease in cell health, a dramatic increase in nonanoic acid was recorded in *D*. *trenchii*. Nonanoic acid is a fatty acid, a group of compounds that can potentially result from increased cell membrane lysis^60,61^. Furthermore, significantly less DMS was detected in *D*. *trenchii* during stress, which was the only compound to significantly decrease in this species. Previous work targeting DMS production in other *Cladocopium* and *Durusdinium* Symbiodiniaceae strains also observed a decrease in DMS with the onset of heat stress^19^. Lower levels of DMS under thermal stress may either indicate that Symbiodiniaceae decrease their production, or that DMS degradation increases due to reactions with harmful molecules in response to thermal stress. Interestingly, in our study, we detected higher amounts of another sulphur compound, dimethyl disulfide (DMDS) in *C*. *goreaui* during stress. DMDS can be formed from the photo-oxidation of methanethiol^62^. These results indicate that we need to consider the full suite of volatile sulphur compounds to fully elucidate the role that these chemicals play in stress response and trophic interactions in coral reefs. An additional 17 compounds significantly increased during thermal stress in *C*. *goreaui*, with 1,3-dimethoxy-2-propanol, UC40.78 and DMDS the main drivers of the differentiation between treatments. Increases in 1,3-dimethoxy-2-propanol may have resulted from lipid peroxidation^63,64^. Whether these BVOCs are released prior to other visual (e.g. bleaching) or physiological^30^ stress indicators remains unknown and will be important to assess for the potential use of specific BVOCs to diagnose early stress responses.

The sheer quantity of detected and unidentified compounds that differed significantly across species and under heat stress highlights a critical need to robustly quantify and classify these BVOCs. However, lack of a comprehensive marine BVOC database severely limits our interpretation of volatilomic data, an issue that similarly limits progress for other metabolomic approaches^33^. Terrestrial BVOC studies have already identified ~30,000 volatile compounds to date^65^. The unidentifiable chemical diversity highlighted here teases at the potential unexplored roles of BVOCs in biological and ecological interactions in marine systems. These unidentified compounds should therefore not be discarded from future analyses as they may play key roles in stress response or could function as useful stress biomarkers that can be measured non-invasively. Volatile databases are continually improving and as such we have made available our mass spectra files (MSV000084436; MassIVE; 10.25345/C5RD5C) for future studies as more comprehensive databases become available.

Numerous BVOCs are known to induce the formation of secondary organic aerosols that enhance cloud formation and albedo (eg. DMS, benzaldehyde, toluene & styrene^66,67^), while other compounds can result in increased formation and residence time of crucial greenhouse gases such as ozone^68^. Marine BVOC emissions are often overlooked when it comes to global modelling of BVOC emissions, largely due to the lack of data from these ecosystems. However, marine ecosystems have a large influence on atmospheric chemistry. Tropical areas are known to have stronger convection forces that lead to greater transport of emitted BVOCs to the troposphere and stratosphere^69^. It is therefore essential to understand current tropical baseline emissions if we wish to accurately model future climate scenarios. Examining the Symbiodiniaceae volatilome is a key step towards understanding the prevalence and function of tropical marine BVOCs. However, further work is needed to clarify how free living Symbiodiniaceae BVOC production varies from endosymbiotic Symbiodiniaceae.

Here we demonstrate for the first time that volatile metabolites produced by Symbiodiniaceae are not only composed of a broad spectrum of BVOCs, but that their production can be influenced by stressful – suboptimal – conditions, suggesting that these overlooked BVOCs likely operate as key constituents regulating metabolic competency. We detected six BVOCs with putative signalling and antioxidant functions that were ubiquitous across the five Symbiodiniaceae genera investigated. Many of the BVOCs reported here are as yet uncharacterised, highlighting an urgent need to develop marine-specific annotation pipelines and to further identify new and abundant compounds. This is of particular relevance given that some of these compounds might play currently uncharacterised roles in resistance and survival of corals during thermal stress events. This work provides direction for future studies to start unravelling the complex functions of volatile metabolites in coral reefs. Corals are some of the most complex symbiotic metaorganisms and the many microbial partners they harbour are likely to contribute to their BVOC emissions. 

## Methods

### Across strain screening of symbiodiniaceae volatilomes

Five isolates, each representing a distinct Symbiodiniaceae genus (*Symbiodinium linucheae*, *Breviolum psygmophilum*, *Durusdinium trenchii*, *Effrenium voratum* and *Fugacium kawagutii*; see Table 1), were maintained in exponential growth in a temperature controlled incubator (Labec; Marrickville, Australia) maintained at 25 °C ± 1.5 °C and under a light intensity of ca. 50 ± 5 μmol photons m^−2^ s^−1^ (HYDRA; Aquaillumination, Ames, Iowa) on a 12:12 light:dark cycle (as per Lawson *et al*.^70^). Cultures were grown in triplicate in 0.2 μm filtered artificial seawater^71^ with IMK medium (Diago, Japan) in sterile 250 mL Schott bottles. Growth and physiological condition of each culture was monitored daily in the five days prior to sampling with direct cell counts and Fast Repetition Rate fluorometry (FRRf; FastOcean, Chelsea Technologies Group, UK). The FRR fluorometer was programmed to deliver single turnover induction of photosystem II (PSII) (i.e. 100 × 1.1 µs flashlets spaced at 2.8 µs intervals) via a blue excitation LED (450 nm). Each acquisition recorded was the mean of 40 consecutive single turnover fluorescent transients, with intervals of 150 ms between acquisitions (detailed in Lawson *et al*.^70^). FRRf measurements were performed on 3 mL live culture, samples were first acclimated to low light (<5 µmol photons m^−2^ s^−1^) for ~15 minutes to relax non-photochemical quenching while simultaneously avoiding build-up of chlororespiration to ensure that only the maximum quantum yield of PSII (*F*_*v*_/*F*_*m*_) was assessed^72^. Cell counts were performed on live cultures using a Neubauer haemocytometer and 20x magnification on a Nikon Eclipse Ci-L compound microscope (Nikon Instruments; Melville, New York). In addition to this daily monitoring data, additional samples were taken on the day of BVOC sampling for FRRf, cell counts, and cell size. For cell size analysis, an aliquot of culture was loaded onto a Neubauer haemocytometer and using a Nikon Upright Fluorescence Microscope (Nikon Instruments; Melville, New York) a series of 48 images were taken for each sample. Using FIJI^73,74^ and White Balance software, these images were processed and the mean cell volume was recorded for each strain^72^. Values of *F*_*v*_/*F*_*m*_ (dimensionless) varied on the day of sampling from 0.412 ± 0.016 (*E*. *voratum*) to 0.479 ± 0.002 (*D*. *trenchii*) (mean ± SE, n = 3; Fig. S2), a range expected across isolates growing in nutrient replete exponential growth^30,72^.

**Table 1:** Symbiodiniaceae isolates examined in the screening experiment. Isolates were maintained in culture and no corals were directly handled in this study. Strains in bold were used for the thermal stress experiment, *indicates use in the screening experiment.

ID	ITS2 Type	Origin	Host	Species
*RT379	A4	Bahamas	*Plexaura homamalla* (Coral)	*Symbiodinium linucheae*
*RT141	B2	Bermuda	*Oculina diffusa* (Coral)	*Breviolum psygmophilum*
**123SCF 058-04**	**C1**	**Magnetic Island (Pacific)**	***Acropora millepora*** **(Coral)**	***Cladocopium goreaui***
*amur-D-MI	**D1a**	**Magnetic Island (Pacific)**	***A***. ***muricata*** **(Coral)**	***Durusdinium trenchii***
*CCMP421	E	Wellington, NZ	Free-living	*Effrenium voratum*
*f156	F1	Hawaii (Pacific)	*Montipora veruccosa* (Coral)	*Fugacium kawagutii*

### Symbiodiniaceae thermal assay experiment

Two Symbiodiniaceae strains characterised by different levels of thermal sensitivity, including the relatively heat sensitive SCF058-04 (*Cladocopium goreaui*) and heat tolerant amur-D-MI (*Durusdinium trenchii*)^75–78^, were subjected to a thermal stress experiment. These isolates were selected based on their differing thermal tolerance and their abundance on the Great Barrier Reef^79,80^. A control incubator (ARALAB; Sintra, Lisboa) was maintained at 26 °C ± 1.5 °C with a light intensity of ca. 100 ± 10 μmol photons m^−2^ s^−1^ (LEDs) on a 12:12 light:dark cycle. A parallel incubator (ARALAB; Sintra, Lisboa) was used for the heat treatment assay with identical settings as for the control, except that temperature was ramped from 26 °C to 30 °C over 4 days and then maintained at 30 °C for 4 days prior to resampling. The temperature was then ramped from 30 °C to 32 °C over 2 days and finally maintained at 32 °C for a further 4 days (Fig. S1). Six biological replicates of each culture were grown (n = 3 per each control and treatment). All cultures were monitored daily with FRRf and cell counts following the same procedures as used in the screening experiment. BVOC sampling was performed at the end of the stress period when the treatment cultures had been at 32 °C for 4 days. Additional samples were taken for cell imaging as per screening experiment on these BVOC sampling days.

### BVOC sampling and volatilome retrieval

Two aliquots of 50 mL from each sample were each placed into a sterile 100 mL crimp cap vial to yield technical duplicate samples with 50 mL head space. Vials were capped and placed in a water bath under light intensity (cool white light, HYDRA; Aquaillumination, Iowa, USA) and temperature that matched their growth or treatment conditions. Samples were purged with instrument grade air (BOC Gases, Linde Group, Australia) for 30 minutes, whereby the purge outlet was passed over thermal desorption (TD) tubes (Tenax TA; Markes International Ltd, Llantrisant, UK; see Fig. S3), which were immediately capped post purge and stored at 4 °C until processing. All TD tubes were analysed within two weeks of sampling by desorbing samples with automated thermal desorption (ULTRA 2 & UNITY 2; Markes International Ltd, Llantrisant, UK) for 6 minutes at 300 °C and concentrated on a Tenax TA cold trap at −30 °C. This cold trap was then flash heated to 300 °C and the concentrated sample injected via a heated transfer line (150 °C) onto a 7890 A GC-MS (Agilent Technologies Pty Ltd, Melbourne) fitted with a BP1 capillary column (60 m × 0.32 mm, 1 µm film thickness; SGE Analytical Science Pty Ltd, Melbourne) at a flow rate of 2.3 mL/minute. Samples were run splitless to allow detection of trace compounds. To allow for complete desorption the GC oven was heated at 35 °C for 5 minutes then 4 °C min^−1^ to 160 °C then 20 °C min^−1^ to 300 °C for 10 minutes. The GC-MS was coupled to a mass-selective detector (Model 5975 C; Agilent Technologies Pty Ltd, Melbourne) that was set to a scanning range of 35–250 amu for the screening experiment and 35–300 amu for the stress experiment. The higher scanning range in the stress experiment was chosen as we examined only two species. The increased scanning range combined with the higher light levels likely led to the higher number of compounds detected in the stress experiment.

Peaks were identified by manually comparing mass spectra against a commercial library (NIST08 library in NIST MS Search v.2.2 f; NIST, Gaithersburg, MD). Blank media samples were run in conjunction with all analysis; the average values for compounds present in the blanks were subtracted from all samples. Common contaminating ions (73, 84, 147, 149, 207 and 221 m/z) were removed using the Denoising function in OpenChrom^81^. Chromatograms were integrated in ChemStation (Agilent Technologies Pty Ltd, Melbourne) with an initial threshold of 14.5 and an initial peak width of 0.068. Files were processed using the MSeasyTkGUI package^82^ in R version 3.5.3 (R Development Core Team, 2015) and all putative compounds clustered. Any compound that did not occur in at least 4 of the 6 replicates (2 technical replicates per biological replicate) was considered contamination and removed. UC denotes an unclassified compound (the number following UC indicates the retention time of the compound if the functional group could not be determined).

### Statistical analyses

A Principal Components Analysis (PCA; Bootstrap N = 1000) on data normalised to total cell volume was completed in the statistical package PAST^83^ to contrast the volatilomes between Symbiodiniaceae species (Screening experiment) and between temperatures (Stress experiment). Compounds were considered “core” components of the volatilome if they appeared in at least 2 out of 3 biological replicates in all species. To test for significant differences between species in the screening experiment, data were processed in MetaboAnalyst4.0, undergoing a generalised logarithm transformation and tested with a one-way ANOVA and Tukey’s HSD post hoc^55,84^. For treatments in the stress experiment, a Kruskal-Wallis test was used (IBM SPSS Statistics, version 25), as data did not meet the assumptions required for parametric tests.

## Supplementary information

Supplementary information

## Data Availability

The datasets generated during the current study are available in the MassIVE database (https://massive.ucsd.edu) under accession numbers: MSV000084436.
