# Supplementary material for: Coral endosymbionts (Symbiodiniaceae) emit species-specific volatilomes that shift when exposed to thermal stress
Source: Sci Rep. 2019 Nov 22;9:17395. doi: 10.1038/s41598-019-53552-0 (PMC6874547; doi:10.1038/s41598-019-53552-0)
Supplement: Supplementary file 1 — Supplementary information [file 41598_2019_53552_MOESM1_ESM.docx]

**Title:**

**Coral endosymbionts (Symbiodiniaceae) emit species-specific volatilomes that shift when exposed to thermal stress**

Authors: Caitlin A. Lawson^1^*, Malcolm Possell^2^, Justin R. Seymour^1^, Jean-Baptiste Raina^1^, David J. Suggett^1^

**Supplementary Material**

**
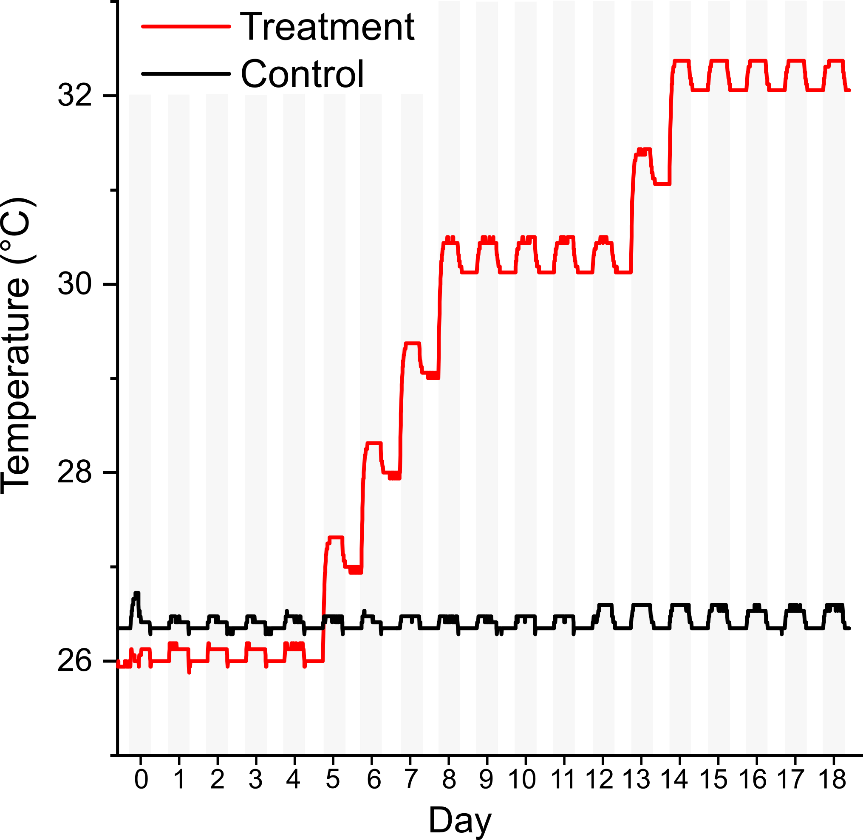
**

**Figure S1.** Temperature profile for the Symbiodiniaceae stress experiment. Light grey shading indicates the light period. Volatiles were sampled on day 18.


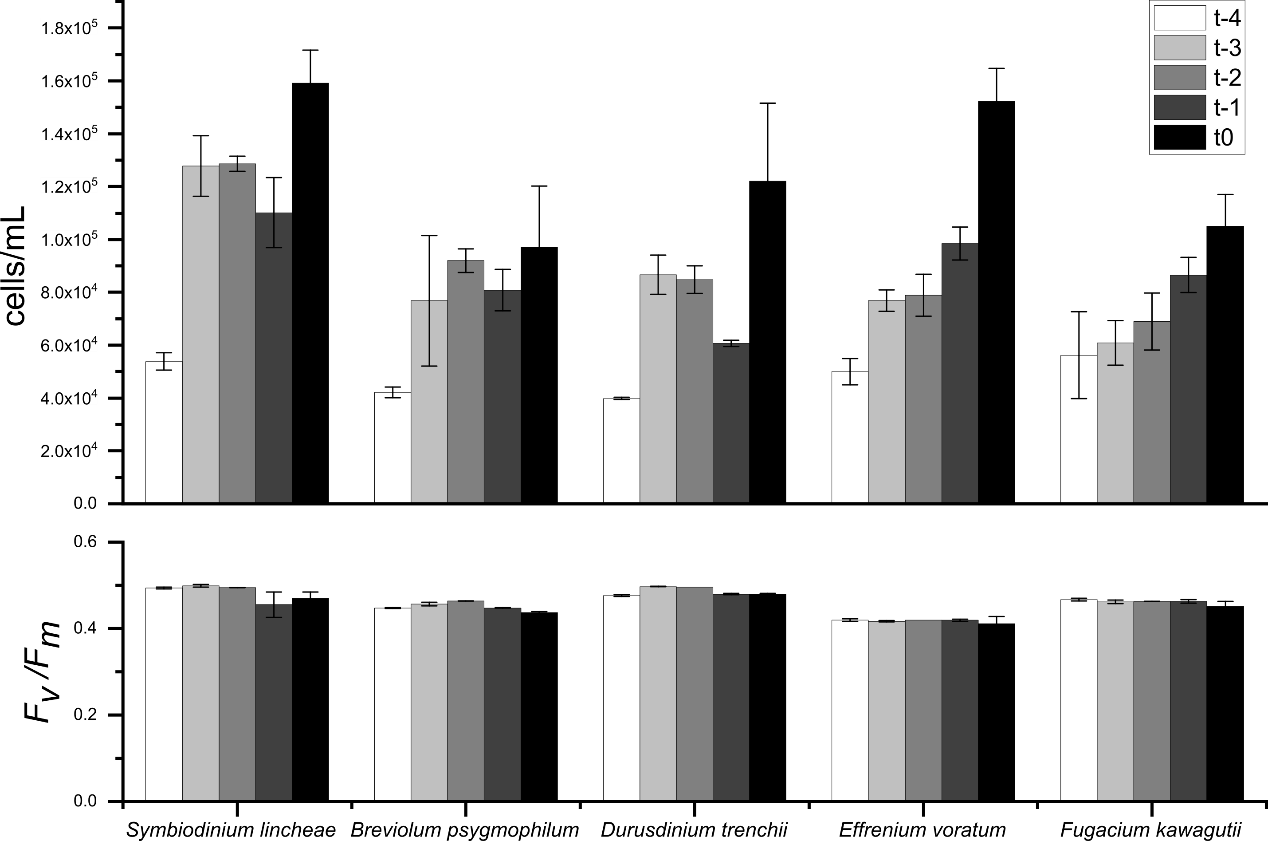


**Figure S2.** Cell density (cells/mL) and F_v_/F_m_ of all Symbiodiniaceae cultures used in the screening experiment in the 4 days prior to BVOC sampling.


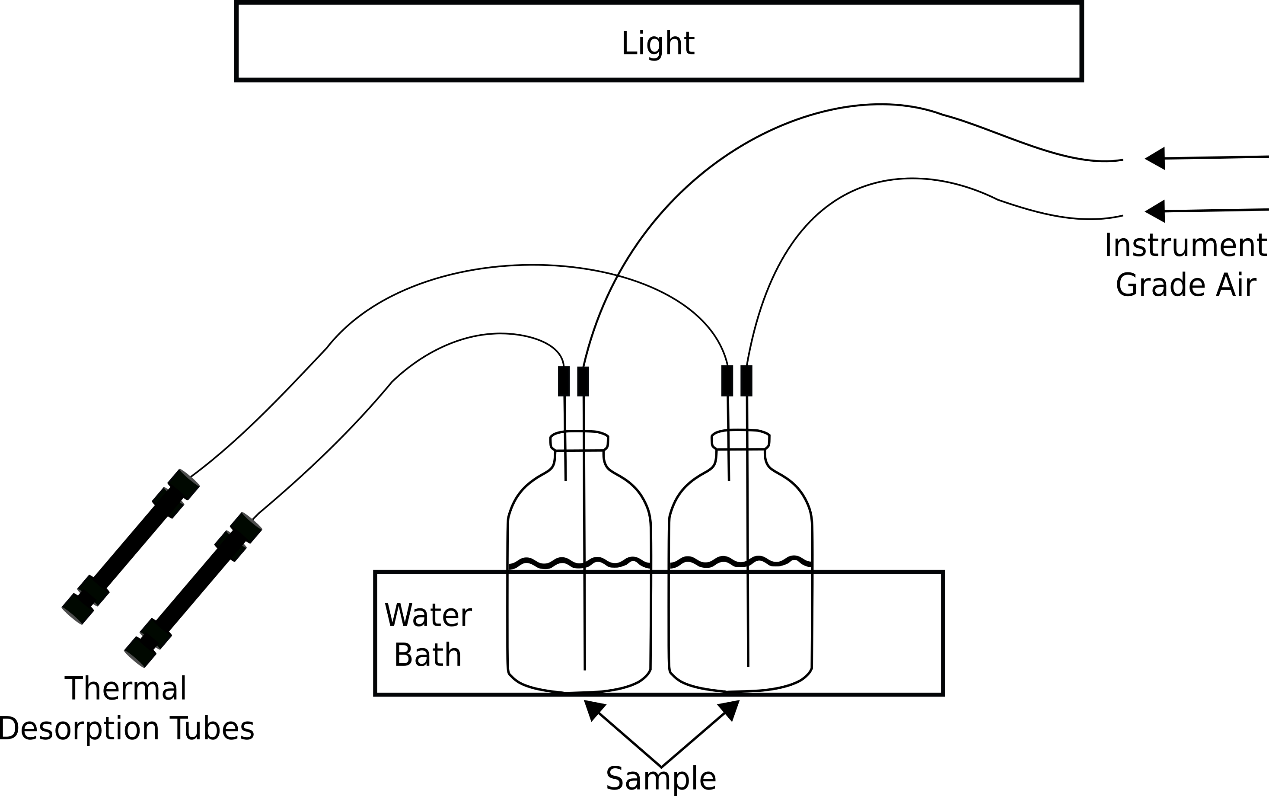


**Figure S3.** Schematic of volatile sampling set up. Culture was placed in gas tight vials and maintained under growth conditions while undergoing a 30 minute purge of instrument grade air. The outlet of this purge passes over Markes thermal desorption tubes (Tenax TA).

**Table S1.** List of all compounds detected in Symbiodiniaceae cultures using the NIST08 library in NIST MS Search v.2.2. DFG = diverse functional groups; UC = Unclassified (the number following UC indicates the retention time of the compound if the functional group could not be determined).

| **SCREENING EXPERIMENT** | | |
| --- | --- | --- |
| Cluster Number | COMPOUND ID | Functional group |
| 13 | UC_18.34 |  |
| 17 | Octadecane, 6-methyl- | Alkane |
| 41 | Halogenated organosilicon | Halogenated organosilicon |
| 50 | Nonanal | Aldehyde |
| 63 | UC 41.89 |  |
| 93 | Benzaldehyde | DFG; aromatic hydrocarbon, aldehyde |
| 94 | Cyclooctyl alcohol | Alcohol |
| 104 | UC alcohol | Alcohol |
| 109 | Squalene | Alkene (Terpenoid) |
| 124 | 3-Trifluoroacetoxypentadecane | Halogenated ester |
| 127 | Styrene | Aromatic hydrocarbon |
| 134 | UC 28.26 |  |
| 139 | UC halogenated hydrocarbon | Halogenated hydrocarbon |
| 146 | 2,4-Dimethyl-1-heptene | Alkene |
| 155 | UC halogenated hydrocarbon | halogenated hydrocarbon |
| 156 | UC_39.59 |  |
| 159 | UC Aldehyde | Aldehyde |
| 162 | UC 40.63 |  |
| 173 | 4-Fluoro-3-trifluoromethylbenzoic acid, eicosyl ester | Halogenated hydrocarbon |
| 185 | Dimethyl sulfide | Organosulfur |
| 186 | Propane, 1,2-dichloro- | Halogenated hydrocarbon |
| 190 | Hexadecane | Alkane |
| 195 | UC Halogenated hydrocarbon | Halogenated hydrocarbon |
| 196 | UC benzoquinone | Ketone |
| 220 | 1-Propanol, 2,2-dimethyl-, benzoate | Ester |
| 226 | UC 40.45 |  |
| 245 | UC 46.85 |  |
| 265 | UC Ester | Ester |
| 269 | Hexane, 2,3-dimethyl- | Alkane |
| 271 | UC Organosulfur | organosulfur |
| 272 | UC ester | Ester |
| 274 | Methyl jasmonate | DFG; aromatic hydrocarbon, carboxylic acid, ketone |
|  |  |  |
| **STRESS EXPERIMENT** | | |
| 1 | l-Alanine ethylamide, (S)- | Amide |
| 11 | Silanol, trimethyl- | Organosilicon |
| 17 | Octadecane, 6-methyl- | Alkane |
| 20 | UC_26.79 |  |
| 24 | 3-Trifluoroacetoxypentadecane | Halogenated ester |
| 25 | Dimethyl disulfide | Organosulfur |
| 29 | 2-Propanol, 1,3-dimethoxy- | DFG; ether, alcohol |
| 33 | UC 24.96 |  |
| 41 | Halogenated organosilicon | Halogenated organosilicon |
| 47 | UC_33.89 |  |
| 50 | Nonanal | Aldehyde |
| 51 | UC Ether | Ether |
| 61 | UC_38.08 |  |
| 62 | UC Ketone | Ketone |
| 64 | Nonanoic acid | Carboxylic acid |
| 71 | UC_36.69 |  |
| 74 | Phenol, 2,4-bis(1,1-dimethylethyl)- | Aromatic hydrocarbon |
| 89 | Toluene | Aromatic hydrocarbon |
| 91 | UC alcohol | Alcohol |
| 95 | UC_33.16 |  |
| 104 | UC alcohol | Alcohol |
| 117 | 1-Pentene, 2,4,4-trimethyl- | Alkene |
| 119 | UC Aromatic hydrocarbon | Aromatic hydrocarbon |
| 120 | UC ester | Ester |
| 125 | Ethylbenzene | Aromatic hydrocarbon |
| 127 | Styrene | Aromatic hydrocarbon |
| 133 | UC 27.81 |  |
| 134 | UC_28.26 |  |
| 136 | Benzene, 1-ethyl-3-methyl- | Aromatic hydrocarbon |
| 137 | UC_30.06 |  |
| 139 | UC halogenated hydrocarbon | Halogenated hydrocarbon |
| 140 | UC 31.50 |  |
| 149 | Azulene | Aromatic hydrocarbon (Monoterpene) |
| 153 | UC_38.58 |  |
| 159 | UC Aldehyde | Aldehyde |
| 160 | 1-Hexadecanol, 2-methyl- | Alcohol |
| 161 | UC_40.78 |  |
| 162 | UC 40.63 |  |
| 166 | UC 42.02 |  |
| 168 | UC Ketone | Ketone |
| 172 | UC 44.07 |  |
| 177 | UC 26.31 |  |
| 178 | UC_39.00 |  |
| 180 | UC 39.36 |  |
| 181 | UC Alkane | Alkane |
| 185 | Dimethyl sulfide | Organosulfur |
| 187 | 2-Butanone, 3,3-dimethyl- | Ketone |
| 193 | Benzene, 1-[1,1-dimethylethyl]-4-[2-propenyloxy]- | Ether |
| 194 | 1-Methoxy-3,5-dimethyl-cyclohexene | Ether |
| 195 | UC Halogenated hydrocarbon | Halogenated hydrocarbon |
| 197 | UC 45.20 |  |
| 203 | Silanediol, dimethyl- | Organosilicon |
| 210 | UC ketone | Ketone |
| 214 | Cyclooctyl alcohol | Alcohol |
| 217 | UC_45.18 |  |
| 225 | Benzoic acid, pentadecyl ester | Ester |
| 236 | Hexane, 2,3-dimethyl- | Alkane |
| 237 | UC_42.46 |  |
| 243 | Heptadecane, 9-octyl- | Alkane |
| 245 | UC 46.85 |  |
| 248 | Oxime-, methoxy-phenyl-_ | DFG, aromatic hydrocarbon, ether, oxime |
| 253 | [2.2]Paracyclophane | Aromatic hydrocarbon |
| 256 | Pentane | Alkane |
| 257 | Naphthalene, 2-methyl- | Aromatic hydrocarbon |
| 264 | UC Alkane | Alkane |
| 265 | US Ester | Ester |
| 267 | UC_42.37 |  |
| 272 | UC ester | Ester |
| 273 | UC 17.25 |  |
| 276 | UC_13.09 |  |
| 278 | UC 38.65 |  |
| 280 | UC_34.06 |  |

**Table S2.** P values for all significant statistical differences detected. The screening experiment one-way ANOVA and Tukey’s HSD post hoc were performed in MetaboAnalyst4.0^54,82^. For the stress experiment analysis, a Kruskal-Wallis test was used (IBM SPSS Statistics, version 25), as data did not meet the assumptions required for parametric tests.

| **Screening experiment** | | |
| --- | --- | --- |
| **Compound ID** | **P value** | **Tukey's HSD** |
| UC_18.34 | 4.34E-12 | B2-A4; D1a-A4; E-A4; F1-A4 |
| Dimethyl sulfide | 1.54E-05 | E-A4; F1-A4; F1-B2; E-D1a; F1-D1a; F1-E |
| 3-Trifluoroacetoxypentadecane | 0.001583 | B2-A4; D1a-A4; E-A4; F1-A4 |
| UC Halogenated hydrocarbon | 0.0025555 | B2-A4; D1a-A4; E-A4; F1-A4 |
| UC_39.59 | 0.003286 | B2-A4; E-A4; D1a-B2; E-D1a |
| **Stress experiment** | | |
| ***Cladocopium goreaui* - testing for differences between control and stress** | | |
| 1,3-Dimethoxy-2-propanol | 0.046 |  |
| 2,4,4-trimethyl-1-pentene | 0.050 |  |
| 2,4-bis(1,1-dimethylethyl)-phenol | 0.050 |  |
| 3,3-dimethyl-2-butanone | 0.050 |  |
| 3-Trifluoroacetoxypentadecane | 0.050 |  |
| 9-Octyl-heptadecane | 0.050 |  |
| Dimethyl disulfide | 0.050 |  |
| Methoxy-phenyl-oxime | 0.050 |  |
| Paracyclophane | 0.050 |  |
| UC Ester | 0.050 |  |
| UC Halogenated hydrocarbon | 0.050 |  |
| UC Ketone | 0.050 |  |
| UC ketone | 0.050 |  |
| UC_13.09 | 0.050 |  |
| UC_34.06 | 0.046 |  |
| UC_40.78 | 0.050 |  |
| UC_42.37 | 0.037 |  |
| UC_42.46 | 0.050 |  |
| ***Durusdinium trenchii* - testing for differences between control and stress** | | |
| 1-Methoxy-3.5-dimethyl-cyclohexene | 0.050 |  |
| 2,4-bis(1.1-dimethylethyl)-phenol | 0.050 |  |
| Benzene 1-[1.1-dimethylethyl]-4-[2-propenyloxy]- | 0.050 |  |
| Benzoic acid pentadecyl ester | 0.037 |  |
| Dimethyl sulfide | 0.050 |  |
| Ethylbenzene | 0.050 |  |
| Nonanal | 0.050 |  |
| Nonanoic acid | 0.046 |  |
| Styrene | 0.050 |  |
| Trimethyl-silanol | 0.050 |  |
| UC alcohol | 0.046 |  |
| UC alcohol | 0.050 |  |
| UC Alkane | 0.050 |  |
| UC halogenated hydrocrabon | 0.050 |  |
| UC Ketone | 0.046 |  |
| UC Ketone | 0.046 |  |
| UC_17.25 | 0.037 |  |
| UC_26.79 | 0.046 |  |
| UC_28.26 | 0.050 |  |
| UC_31.50 | 0.050 |  |
| UC_33.89 | 0.037 |  |
| UC_36.69 | 0.050 |  |
| UC_38.58 | 0.050 |  |
| UC_40.78 | 0.050 |  |
| UC_44.07 | 0.037 |  |
